# Knockdown of NCAPD3 inhibits the tumorigenesis of non-small cell lung cancer by regulation of the PI3K/Akt pathway

**DOI:** 10.1186/s12885-024-12131-x

**Published:** 2024-04-02

**Authors:** Fan Yang, Yunfeng Zheng, Qiong Luo, Suyun Zhang, Sheng Yang, Xiangqi Chen

**Affiliations:** 1 Department of Respiratory Medicine, Fujian Medical University Union Hospital, NO.29 of Xinquan Road, Gulou District, 350000 Fuzhou City, Fujian Province China; 2 Department of Gastric Surgery, Fujian Medical University Union Hospital, NO.29 of Xinquan Road, Gulou District, 350000 Fuzhou City, Fujian Province China; 3 Department of Oncology, Fujian Medical University Union Hospital, NO.29 of Xinquan Road, Gulou District, 350000 Fuzhou City, Fujian Province China; 4 Department of Internal Medicine, Fujian Medical University Union Hospital, 350001 Fuzhou, Fujian China

**Keywords:** NCAPD3, Non-small cell lung cancer, PI3K/Akt/FOXO4, Proliferation, Apoptosis

## Abstract

**Background:**

Accumulating evidence indicates that aberrant non-SMC condensin II complex subunit D3 (NCAPD3) is associated with carcinogenesis of various cancers. Nevertheless, the biological role of NCAPD3 in the pathogenesis of non-small cell lung cancer (NSCLC) remains unclear.

**Methods:**

Immunohistochemistry and Western blot were performed to assess NCAPD3 expression in NSCLC tissues and cell lines. The ability of cell proliferation, invasion, and migration was evaluated by CCK-8 assays, EdU assays, Transwell assays, and scratch wound healing assays. Flow cytometry was performed to verify the cell cycle and apoptosis. RNA-sequence and rescue experiment were performed to reveal the underlying mechanisms.

**Results:**

The results showed that the expression of NCAPD3 was significantly elevated in NSCLC tissues. High NCAPD3 expression in NSCLC patients was substantially associated with a worse prognosis. Functionally, knockdown of NCAPD3 resulted in cell apoptosis and cell cycle arrest in NSCLC cells as well as a significant inhibition of proliferation, invasion, and migration. Furthermore, RNA-sequencing analysis suggested that NCAPD3 contributes to NSCLC carcinogenesis by regulating PI3K/Akt/FOXO4 pathway. Insulin-like growth factors-1 (IGF-1), an activator of PI3K/Akt signaling pathway, could reverse NCAPD3 silence-mediated proliferation inhibition and apoptosis in NSCLC cells.

**Conclusion:**

NCAPD3 suppresses apoptosis and promotes cell proliferation via the PI3K/Akt/FOXO4 signaling pathway, suggesting a potential use for NCAPD3 inhibitors as NSCLC therapeutics.

**Supplementary Information:**

The online version contains supplementary material available at 10.1186/s12885-024-12131-x.

## Introduction

Lung cancer is the most lethal malignant tumor worldwide, and its incidence and mortality rates are growing annually [1]. Lung adenocarcinoma (LUAD) and lung squamous cell carcinoma (LUSC) are the most prevalent subtypes of non-small cell lung cancer (NSCLC), accounting for approximately 85% of lung cancer [2]. Clinically, NSCLC is frequently diagnosed in advanced stages, with an extremely low five-year survival rate ranging from only 10–20% [1]. The advent of immune checkpoint inhibitors (ICIs) and molecular-targeted therapy has significantly improved the prognosis of patients with advanced-stage cancer, particularly those with NSCLC [3, 4]. Despite the potent anti-cancer activity of these therapies, approximately 50% of pre-treated patients do not derive any benefit, with a small subgroup experiencing early progression or death within 3 months of treatment [5]. Given the complexity of pathobiology and the emergence of drug resistance, which requires the further unravelling of molecular mechanisms and the development of innovative therapeutic strategies.

Non-SMC condensin II complex subunit D3 (NCAPD3) belongs to the chromosome condensin II complex, which plays a crucial role in chromosome assembly and segregation during mitosis and meiosis [6]. In addition to its functions in chromosome condensation and segregation, recent research has revealed that NCAPD3 is involved in the regulation of cancer progression. NCAPD3 is highly expressed in pancreatic ductal adenocarcinoma and associated with reduced overall survival [7]. Furthermore, NCAPD3 has been identified as a novel biomarker for subtype-1 prostate cancer [8]. In colorectal cancer, NCAPD3 promotes tumor progression by enhancing the Warburg effect [9]. However, the role of NCAPD3 in NSCLC and its molecular mechanisms remains unclear.

In present study, we demonstrated that the expression of NCAPD3 was upregulated in NSCLC tissues and cell lines. We further elucidated the importance of NCAPD3 in terms of prognosis and clinicopathology in NSCLC. Specifically, our findings revealed that NCAPD3 functions as an oncogene, promoting the proliferation of NSCLC cells while inhibiting apoptosis via the PI3K/AKT/FOXO4 signaling pathway.

## Materials and methods

### Oncomine database

To accelerate the discovery process in gene-wide expression investigations, the Oncomine database (http://www.oncomine.org) serves as an online cancer microarray database for tumor-related analysis [10, 11]. The mRNA levels of NCAPD3 in NSCLC were determined through analysis of the ONCOMINE including three separate datasets (Hou lung [12], Beer lung [13], and Stearman lung [14]). The thresholds were ser as follows: *p* = 0.01; fold change = 1.5; and mRNA data type.

### UALCAN data analysis

UALCAN (http://ualcan.path.uab.edu) is a comprehensive webportal for TCGA transcriptome data analysis [15]. In our research, we investigated the NCAPD3 expression in normal and NSCLC tissues according to histological type (categorized as LUAD or LUSC) and different tumor stages (classified as stage I-IV). Wherein, cancer stage I denotes that localized cancer confined to the primary site; stage II represents that cancer spread to regional lymph nodes; stage III indicates cancer spread to adjacent tissues; and stage IV indicates further spread to distant organs [16].

### Patient samples

Paraffin-embedded tumor tissue and adjacent non-tumor tissue from 184 NSCLC patients between 2012 and 2015 were collected from the Department of Pathology of Fujian Medical University Union Hospital. In the present study, none of the patients had received preoperative chemotherapy, radiation therapy, or adjuvant therapies. The application of archived cancer samples and clinical data was approved by the Ethics Committee of Fujian Medical University Union Hospital (Ethics Approval number: 2020WSJK016).

### Immunohistochemistry

Immunohistochemistry was performed on paraffin-embedded human NSCLC specimens using anti-NCAPD3 (1:1000; Abnova Inc., Taiwan, China) mouse monoclonal antibody. Briefly, deparaffinization, rehydration, antigen extraction, IHC labeling, and pathology scoring were used to process samples. Based on IHC, two pathologists assess the expression of NCAPD3 based on the immunoreactivity score (IRS). Scoring factors included the percentage of tumor cells that stained positively and the intensity of staining. Five categories of positive staining were assessed and determined: 5% positive cells for a score of 0; 5 to 25% for 1; 26 to 50% for 3; and 76% for 4. Scores for stain intensity ranged from 0 (no staining) to 1 (light yellow), 2 (yellow), or 3 (brown). Low expression (≤ 4) and high expression (> 4) were considered based on the intensity and proportion score [17]. Images were collected under microscope.

### Cell culture and lentiviral infection

BEAS-2B and human NSCLC cell lines A549, H1975, H1299 and SPCA-1 were acquired from the Cell Bank of the Chinese Academy of Science (Shanghai, China). NCAPD3 interference lentivirus (shNCAPD3) and control lentivirus (shNC) were generated by Genechem Co., LTD (Shanghai, China). For NCAPD3 interference research, SPCA-1 and A549 cells were employed. Additionally, the optimal multiplicity of infection was determined, and subsequently, infected cells were selected by puromycin. Ultimately, the expression of NCAPD3 was assessed by RT-qPCR and Western blot. The shRNA sequences are provided in Supplementary Table 1.

### Real-time quantitative PCR (RT-qPCR)

Total RNA was extracted using TRIzol reagent (Takara,Japan). Reverse transcription was performed with the PrimeScript RT reagent Kit (Takara). mRNA levels were measured with gene-specific primer using the SYBR Green PCR Kit (Thermo Fisher Scientific, MA, USA). The relative expression of target mRNA was analyzed by the 2^−ΔΔCt^ method. The primers for RT-qPCR were provided in Supplementary Table 2.

### Western blot

Total protein was extracted with RIPA buffer (Solarbio, Beijing, China) for 40 min before being centrifuged (12,000 rpm, 10 min, 4 °C). The isolated proteins were then transferred to a PVDF membrane after being separated using an 8-15% SDS-PAGE gel (Millipore, MA, USA). The membranes were treated with primary antibodies and then incubated for one hour at room temperature with the second mouse- or rabbit-specific antibody for each primary antibody. The Bio-Rad ChemiDocTMXRS system and conventional chemiluminescence were used to identify the blots at the end. Detailed antibody information and dilution ratios were provided in Supplementary Table 3.

### Cell proliferation assays

3 × 10^3^ NSCLC cells/well were injected into 96-well plates and applied 10ul CCK-8 reagent (Beyotime, Tianjin, China) into each well at 0 h, 24 h, 48 h, and 72 h, respectively. The absorbance value (OD) at 450 nm was evaluated by a microplate reader (Bio-Rad, CA, USA).

In term of the EdU assay, 1 × 10^4^ cells/well were inoculated in 24-well plates, and then cultured with 250 µL EdU medium and Apollo staining solution (RiboBio, China). The positive cells were then quantified using a fluorescence microscope via counting at least 5 random fields.

### Transwell invasion assays

NSCLC cells (1 × 10^5^) were plated into the upper chamber of the 24-well transwell insert chambers (Corning, NY, USA). Chambers were precoated with 30 ul Matrigel (BD Bioscience, CA, USA), which was diluted with serum-free medium (ratio of 1:8) and then incubated at 37 ℃ for 1 h. After incubation for 48 h, cells that migrated to the lower chamber were fixed with 4% paraformaldehyde (Biosharp, China) for 20 min and stained with 0.1% crystal violet (Solarbio Lifer Science, China) for 10 min. Five randomly chosen visual fields were counted and imaged with the microscope.

### Scratch wound healing assays

5 × 10^5^ cells were seeded in 6-well plates with complete culture medium and then grown for overnight. When NSCLC cells proliferated to 90% confluence, a 200ul sterile plastic tips was used to scratch a wound line. The suspension cells were thoroughly washed with PBS, and cultured in 2 ml serum-free medium at 37 ℃ for 48 h. Representative images at the indicated time were taken under the microscope in three random fields. The wounds were assessed using Image J.

### Flow cytometry

Cells were cultured in 6-well plates overnight. For cell apoptosis assay, cells were collected and resuspended in 195 µl binding buffer, then 5 µl Annexin V-FITC and 10 µl PI (Beyotime, China) were added. After incubating at room temperature for 30 min, the samples were measured by flow cytometer (Beckman Coulter, USA).

For cell cycle analysis, the cells were fixed with 70% ethanol overnight, then stained with PI for 30 min, and finally analyzed using flow cytometry.

### RNA sequencing assay and data analysis

RNA sequencing was performed by SeqHealth Technology Company (Wuhan, China). Total RNAs were extracted from NSCLC cells with or without NCAPD3 knockdown using TRIzol Reagent (Takara, Japan). After confirming the quality and integrity of RNA, 2ug RNAs were used for standard RNA sequencing library preparation, and the products were sequenced on Novaseq 6000 sequencer (Illumina). The detailed protocols and data analysis were recorded in Supplementary File 1. The expression data is accessible in the Gene Expression Omnibus data repository (GEO) under the accession number GSE249569.

### Statistical analysis

All experiments were performed in triplicate, Statistical analyses were performed using GraphPad Prism 6 software (San Diego, CA, USA). Values were statistically examined using a one-way ANOVA analysis or Student’s t-test (two-tails), and are presented as the mean ± SEM. *P* values < 0.05 were considered statistically significant [18].

## Results

### NCAPD3 is highly expression in NSCLC patients and correlates with poor prognosis

**Fig. 1:**
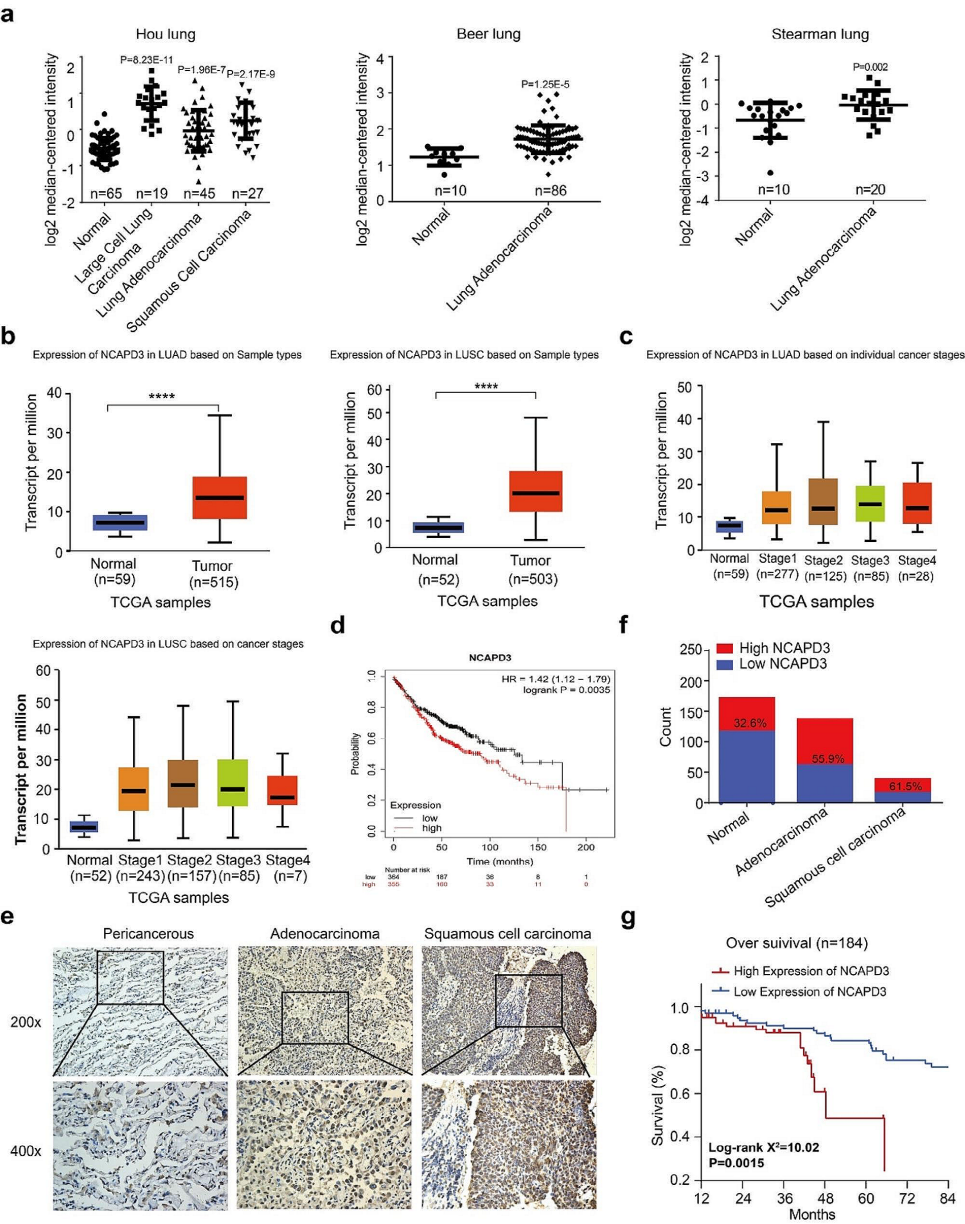
NCAPD3 was overexpressed in NSCLC and associated with worse prognosis. **a** In Hou lung, Beer lung, and Stearman lung Oncomine datasets, NCAPD3 was upregulated in NSCLC tissues as compared with normal lung tissues. **b** UALCAN analysis of NCAPD3 expression in TCGA database. **c** NCAPD3 expression in LUAD and LUSC patients at different cancer stages from UALCAN database. **d** Kaplan-Meier Plotter website analyzing the correlation between NCAPD3 and survival in NSCLC patients. **e** Representative images of NCAPD3 expression in LUAD and LUSC tissues based on IHC. **f** The percentage of NSCLC tissues and para-carcinoma normal tissues exhibiting high ( + + or +++) and low (- or +) expression of NCAPD3. **g** Kaplan-Meier survival curves for OS in high or low NCAPD3 expression groups; low expression group, *n* = 75; high expression group, *n* = 109. (****p < 0.0001)

To assess the expression level of NCAPD3 mRNA in NSCLC, we analyzed three independent NSCLC microarray datasets on Oncomine and discovered that NSCLC exhibited a higher level of NCAPD3 mRNA compared to normal lung tissues (*p <* 0.05) (Fig. 1a). Additionally, we also examined the expression of NCAPD3 in the UALCAN online tumor database. Consistent with our findings, the UALCAN database demonstrated a significant upregulation of NCAPD3 expression in both LUAD and LUSC tissues compared to normal lung tissues (*p <* 0.05) (Fig. 1b). Furthermore, we explored the expression level of NCAPD3 across different tumor stages for LUAD and LUSC patients. Interestingly, the expression exhibited an upward trend with increasing tumors stages (Fig. 1c). Kaplan-Meier curves were generated using data from The Cancer Genome Atlas (TCGA) database to assess the effect of NCAPD3 gene on the prognosis of NSCLC. The survival curve analysis revealed that higher expression of NCAPD3 was related to worse OS (HR = 1.42, log-rank *p <* 0.05) (Fig. 1d).

Immunohistochemical staining was performed to assess the clinical relevance of NCAPD3 expression in 184 human NSCLC specimens. Comparing to adjacent non-cancerous tissues, the expression of NCAPD3 greatly upregulated in LUAD and LUSC tissues (Fig. 1e, f). Further analyses revealed a positive correlation between the NCAPD3 staining and tumor-node-metastasis (TNM) stage and grade (*p <* 0.05), but not with age, gender, and histology (*p >* 0.05) (Table 1). Additionally, we conducted a Kaplan-Meier survival analysis of the 184 lung cancer patients according to their NCAPD3 expression levels to investigate whether NCAPD3 is a possible prognostic factor for lung cancer. Based on the intensity of IHC staining, we classified tissue samples as either having negative (*n* = 75) or positive (*n* = 109) NCAPD3 expression. Kaplan-Meier analysis revealed that higher NCAPD3 expression in lung cancer patients was associated with a worse clinical outcome compared to low expression (log-rank *p* = 0.0015) (Fig. 1g). These findings revealed that NCAPD3 was abnormally high expressed in NSCLC tissues, and the cases in the high expression group had a poorer prognosis reflected by OS.

**Table 1:** Relationship between NCAPD3 gene and clinicopathological variables of NSCLC

Characteristics	No. of patients	NCAPD3 protein expression		χ^2^	p-value
		Low	High		
Age				0.67	0.41
< 60	89	39	50		
≥ 60	95	36	59		
Gender				3.31	0.07
Female	81	27	54		
Male	103	48	55		
Grade				-	< 0.05
I + II	150	74	76		
III + IV	34	1	33		
T stage				107.16	< 0.05
T1	92	72	20		
T2 + 3 + 4	92	3	89		
N stage				13.08	< 0.05
N0	133	65	68		
N1 + 2	51	10	41		
Histology				1.13	0.28
LUAD	145	62	83		
LUSC	39	13	26		

Abbreviations: *LUAD*, lung adenocarcinoma; *LUSC*, lung squamous cell carcinoma

Table 1 Relationship between NCAPD3 gene and clinicopathological variables of NSCLC.

**Table:** 

Characteristics	No. of patients	NCAPD3 protein expression		χ^2^	p-value
		Low	High		
Age				0.67	0.41
< 60	89	39	50		
≥ 60	95	36	59		
Gender				3.31	0.07
Female	81	27	54		
Male	103	48	55		
Grade				-	< 0.05
I + II	150	74	76		
III + IV	34	1	33		
T stage				107.16	< 0.05
T1	92	72	20		
T2 + 3 + 4	92	3	89		
N stage				13.08	< 0.05
N0	133	65	68		
N1 + 2	51	10	41		
Histology				1.13	0.28
LUAD	145	62	83		
LUSC	39	13	26		

*LUAD*, lung adenocarcinoma; *LUSC*, lung squamous cell carcinoma.

### NCAPD3 knockdown inhibits the proliferation, invasion and migration in NSCLC cells

**Figure 2:**
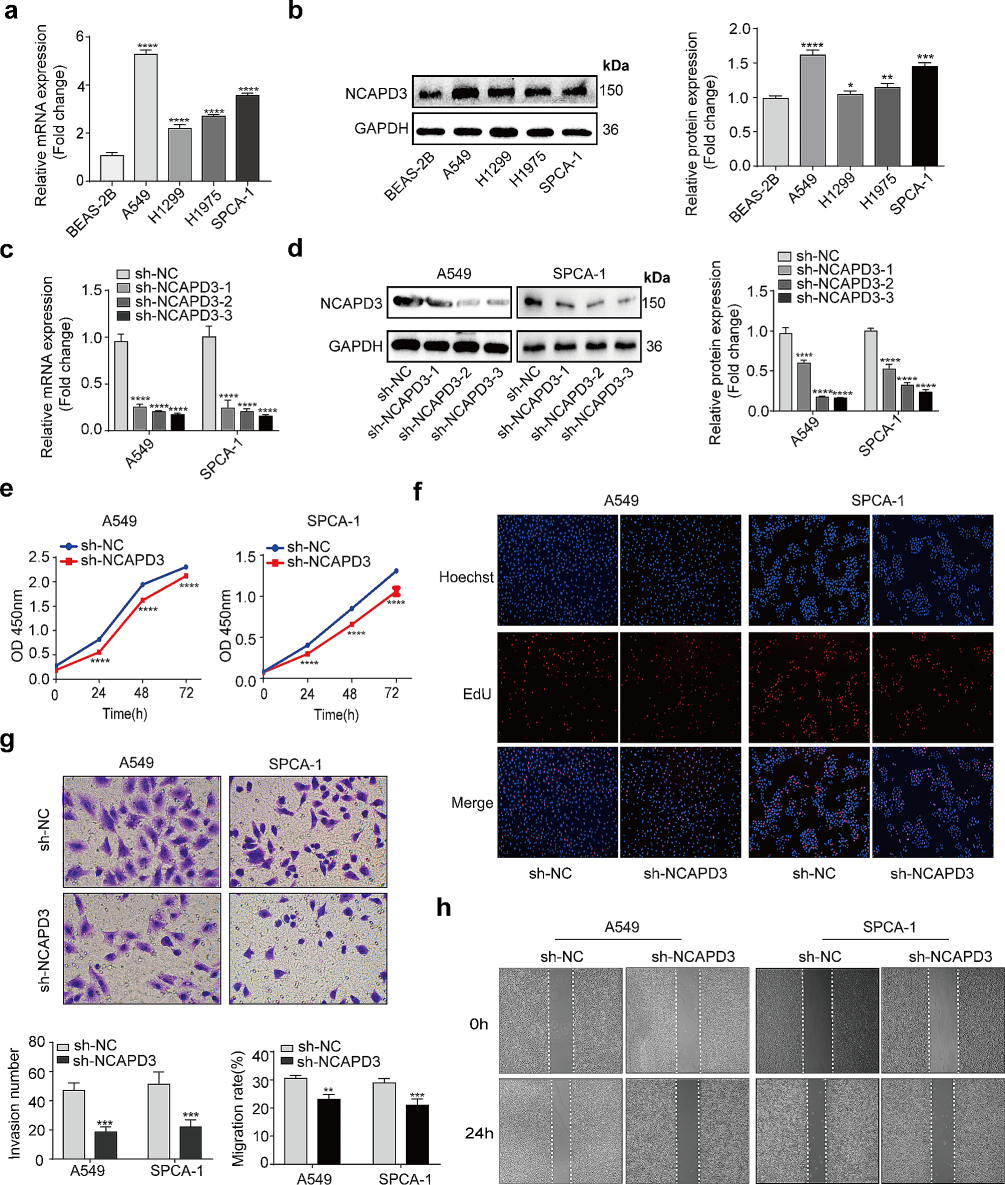
Knockdown of NCAPD3 inhibited NSCLC cell proliferation and invasion. **a-b** The expression of NCAPD3 in NSCLC cell lines was assessed through RT-qPCR and Western blot. **c-d** The knockdown efficiency was detected through RT-qPCR and Western blot in A549 and SPCA-1 cells by transfecting with specific shRNAs. **e** The effect of NCAPD3 on cell proliferation was measured using CCK-8 assays. **f** The proliferation of cells was determined by EdU assays. **g** Transwell analysis of the invasive capacity of A549 and SPCA-1 cells. **h** Migration capacity was evaluate by wound-healing assays; all results were expressed as the means ± SD. (***p < 0.001; ****p < 0.0001)

To investigate the impact of NCAPD3 on NSCLC, we assessed the mRNA and protein expression of NCAPD3 in normal lung epithelial cell line (BEAS-2B) and multiple NSCLC cell lines (A549, H1299, H1975, and SPCA-1) by RT-qPCR and Western blot. Our results were in agreement with our observations in NSCLC specimens, indicating the expression of NCAPD3 was significant upregulated in lung cancer cell lines, especially in the A549 and SPCA-1 (Fig. 2a, b). Next, we transfected three NCAPD3 shRNAs into A549 and SPCA-1 cells, and evaluated their knockdown efficiency through qRT-PCR and Western blot (Fig. 2c, d). The function of shRNAs on cell proliferation was assessed through CCK-8 assays and EdU assays (Fig. 2e, f). The findings demonstrated that a significant decrease in proliferation in shNCAPD3-transfected A549 and SPCA-1 cells compared to cells transfected with shNC. Transwell tests and scratch assays were used to further examine how NCAPD3 affected cell invasion and motility (Fig. 2g, h). Furthermore, knockdown of NCAPD3 greatly inhibited the migratory and invasive capacity of NSCLC cells, which is consistent with the finding that lower NCAPD3 expression is correlated to a reduced risk of lymph node metastasis and a higher overall survival rate.

### NCAPD3 knockdown induces G0/G1 cell cycle arrest and enhances apoptosis

**Figure 3:**
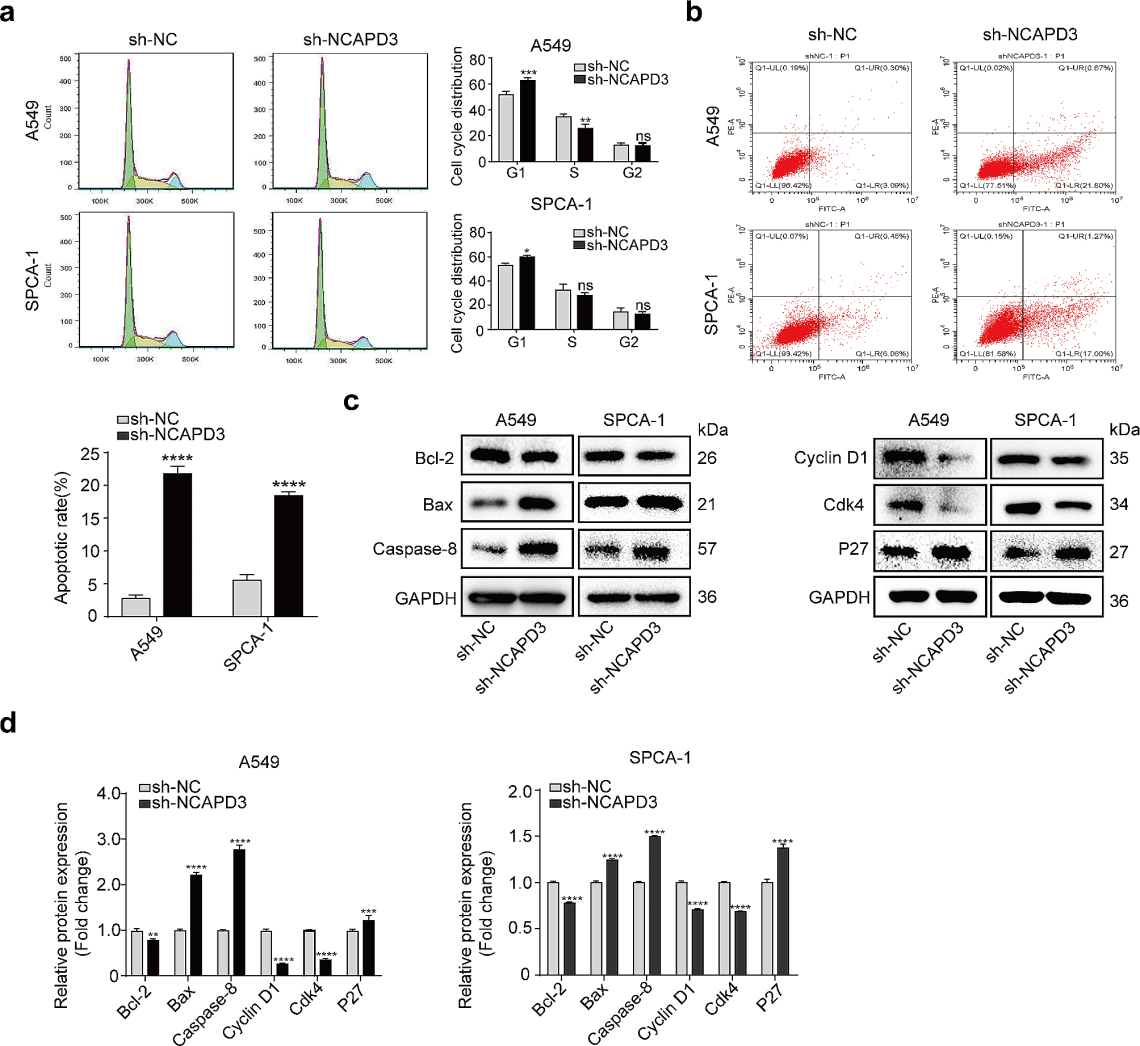
Effects of NCAPD3 knockdown on the cell cycle and apoptosis of NSCLC cells. **a-b** Flow cytometry assays were performed to assess the impact of NCAPD3 on the cell cycle and apoptosis. **c-d** The expression of apoptosis-related markers (Bcl-2, Bax, and Caspase-8) and cell cycle-related markers (Cyclin D1, Cdk4, and P27) were evaluated by Western blot. (*p < 0.05, ***p < 0.001, ****p < 0.0001, ns: not significant)

We examined the effect of NCAPD3 knockdown on the cell cycle distribution in A549 and SPCA-1 cells using flow cytometry. Compared to control cells, the proportion of G0/G1 phase cells greatly upregulated in the sh-NCAPD3 group, while S phase cells dramatically reduced (Fig. 3a). Then, flow cytometry analysis of Annexin VeFITC/PI was conducted to determine the impact of NCAPD3 on cell apoptosis. The results revealed that NCAPD3-targeted shRNAs cell lines had a greatly higher proportion of apoptotic cells compared to the shNC groups (Fig. 3b)

Furthermore, to better understand the molecular process underlying the effect of NCAPD3 on the cell cycle and apoptosis in NSCLC cells, we investigated the key regulators of G0/G1 phase transition (including Cyclin D1, Ckd4, and P27) and apoptosis-related markers (including Bcl-2, Bax and Caspase-8) for detection. Our findings illustrated that NCAPD3 knockdown suppressed the expression of Cyclin D1, and Ckd4, which are known to regulate the G0/G1 phase transition. While the expression of P27 was obviously increased (*p* < 0.05). Furthermore, Western blot analysis also indicated that as compared to control groups, the expression of Bcl-2 was significantly downregulated, while Bax and Caspase-8 were up-regulated in NCAPD3 knockdown cells (Fig. 3c, d)

### NCAPD3 knockdown inhibits proliferation and enhances apoptosis by regulation of the PI3K/AKT pathway

**Figure 4:**
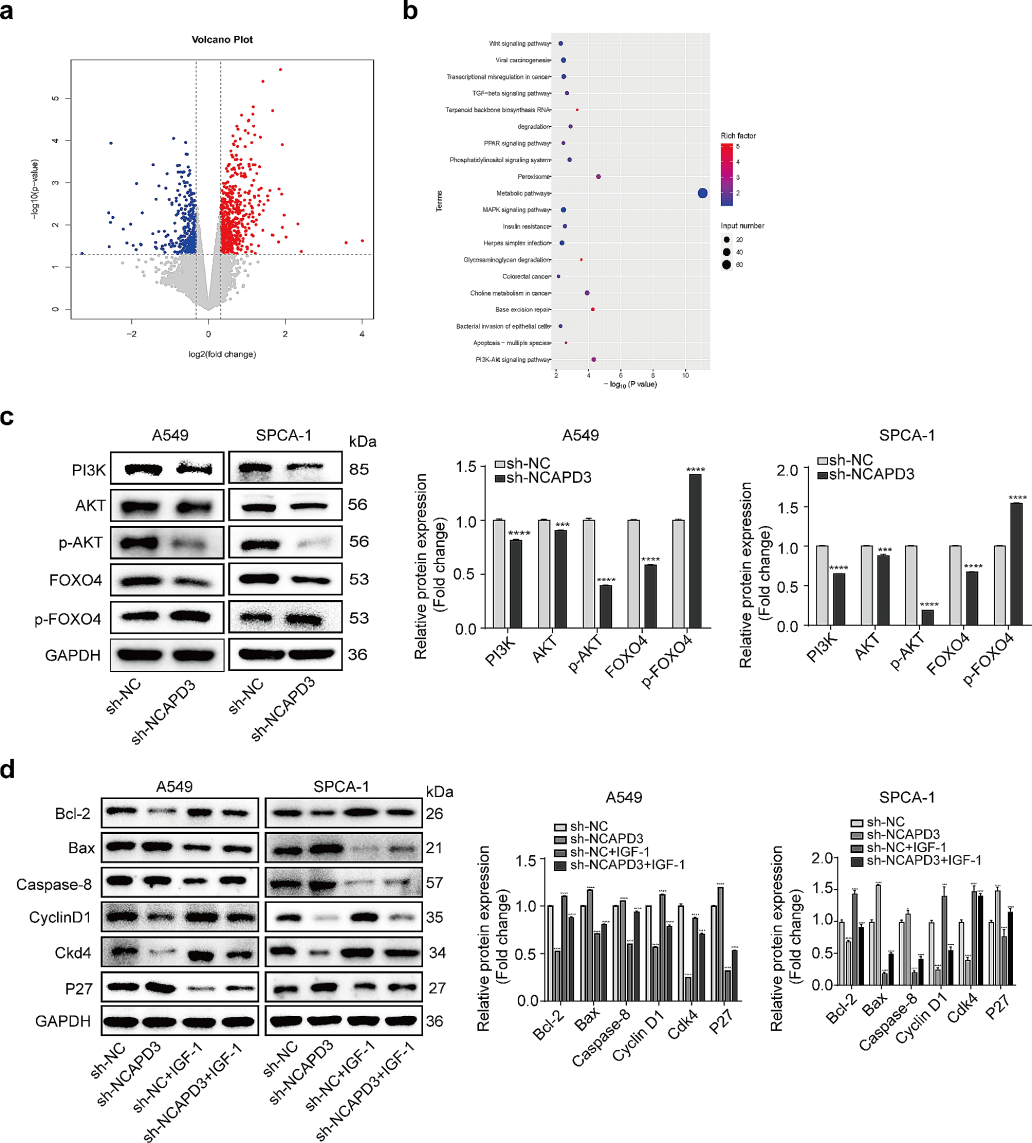
Knockdown of NCAPD3 suppressed NSCLC cell proliferation by PI3K/AKT/FOXO4 signaling. **a** A volcano scatterplot illustrated the differentially expressed genes (DEGs). Blue dots show downregulated genes by NCAPD3 knockdown, grey dots represent unaltered genes, and red dots represent upregulated genes. (|Fold of changes| >1, *p* value < 0.05). **b** KEGG enrichment analysis was performed to investigate the downstream signaling pathway by identifying DEGs [19, 23]. **c** PI3K/AKT/FOXO4 components in A549 and SPCA-1 cells were assessed by Western blot. **d** Western blot was conducted to examine cell apoptosis-related markers (Bcl-2, Bax, and Caspase-8) and cell proliferation-related markers (Cyclin D1, Cdk4 and P27) in infected A549 and SPCA-1 cells treated with IGF-1 (100 ng/mL) for 24 h. (***p < 0.001, ****p < 0.0001)

To explore the underlying mechanism of NCAPD3 in regulating NSCLC progression, we employed a transcriptome sequencing approach to compare A549 cells transfected with shNCAPD3 to shNC-transfected cells. Compared with the control group, there are 1484 differentially expressed genes (DEGs) were significantly regulated upon NCAPD3 downregulation (|log2(fold change)| > 1, *p* value < 0.05), and included 876 upregulated genes and 608 downregulated genes (Fig. 4a). To further analyzed the biological functions and signaling pathways of the DEGs, GO and KEGG enrichment analyses were performed [19, 20]. KEGG pathway analysis revealed that the top enriched pathways were metabolic pathways, peroxisome, and PI3k-AKT signaling pathway (Fig. 4b). Those findings demonstrated that NCAPD3 may contribute to cell survival via modulating the PI3K/AKT signaling pathway

 Furthermore, our study further confirmed the hypothesis through Western blot analysis. The findings demonstrated that PI3K and p-AKT expression was reduced, while p-FOXO4 was upregulated when NCAPD3 silenced (Fig. 4c). Moreover, to further support the involvement of the PI3K/AKT signaling pathway in the oncogenic effect of NCAPD3, A549 and SPCA-1 cells transfected with shNCAPD3 or shNC were treated with 100 ng/ml insulin-like growth factors-1 (IGF-1), reported as one of PI3K/AKT signaling activator [21, 22]. The expression of cell cycle and apoptosis biomarkers were then assessed via Western blot. These findings revealed that IGF-1 could prevent the effects of NCAPD3 silencing on cell proliferation and apoptosis (Fig. 4d). Together, these findings suggest that the PI3K/AKT/FOXO4 pathway may play a crucial role in mediating the carcinogenic effects of NCAPD3 on NSCLC cell proliferation and its effects on preventing apoptosis

## Discussion

Uncontrolled proliferation is one of the hallmarks of cancer. Under abnormal conditions, mutations in genes that regulate the cell cycle, proliferation and division can lead to abnormal cell proliferation processes, allowing tumors to exceed the boundaries of normal tissue growth [7]. Apoptosis can occur in a variety of physiological and pathological conditions and is a unique intrinsic cellular death program regulated by multiple genes [24]. The balance between cell proliferation and apoptosis is necessary for tissue homeostasis [7]. The regulation of proliferation and inhibition of apoptosis is the essence of all tumorigenesis, and they also provide opportunities for therapeutic intervention in cancer. Related studies have revealed a strong correlation between condensin complex subunits, responsible for chromosome condensation and segregation throughout the cell cycle, and the progression of tumors. Condensin complexes I and II are essential for chromatin condensation and stability in prophase mitosis. Abnormal expression of complex subunits will incomplete condensation of chromatin [25]. Previous research has demonstrated that poor prognosis is correlated to abnormal condensin complex I expression of the NCAPH, NCAPG, and NCAPD2 subunits in NSCLC, liver cancer, colorectal cancer, breast cancer, and prostate cancer [26–31]. Meng et al. found that knockout of the NCAPG2 subunit of condensin complex II greatly reduced the proliferation, invasion and migration of hepatocarcinoma cells, and caused G1/S phase arrest [32]. Whereas, the biological process and potential mechanisms of NCAPD3 in NSCLC remain unknown

In the present study, we identified NCAPD3 as a potential gene involved in the development of NSCLC using bioinformatics technology. Subsequently, we conducted further investigations to examine the elevated expression of NCAPD3 in NSCLC tissues through immunohistochemical staining. Additionally, we successfully established NCAPD3 knockdown lung cancer cell lines and performed in vitro functional assays to investigate the regulatory role of NCAPD3 on the biological behavior of NSCLC cells. These results demonstrated that the knockdown of NCAPD3 expression can dramatically suppress the proliferation, invasion and migration of NSCLC cells. Subsequently, we investigated whether NCAPD3 can modulate the cell cycle and apoptosis of NSCLC cells. And our results revealed that downregulation of NCAPD3 expression significantly increased the number of apoptotic cells, elevated the proportion of cells in the G1 phase, and induced G1/S phase arrest. Considering all of our findings, our results demonstrated that NCAPD3 may function as an oncogene and play a crucial role in the development and progression of NSCLC.

Our research investigated the potential mechanism by which NCAPD3 affects apoptosis and cell cycle regulation in NSCLC, focusing on the expression of cell cycle and apoptosis-related proteins. Our findings demonstrated that NCAPD3 gene suppressed tumor cell apoptosis through up-regulation anti-apoptotic protein Bcl-2, while down-regulating pro-apoptotic proteins including caspase-8 and Bax, ultimately inactivating the apoptotic cascade in tumors. Moreover, down-regulating NCAPD3 resulted in the suppression of CyclinD1 and CKD4 expression, while up-regulation of the inhibitory protein P27, contributing to G1/S phase arrest

**Figure 5:**
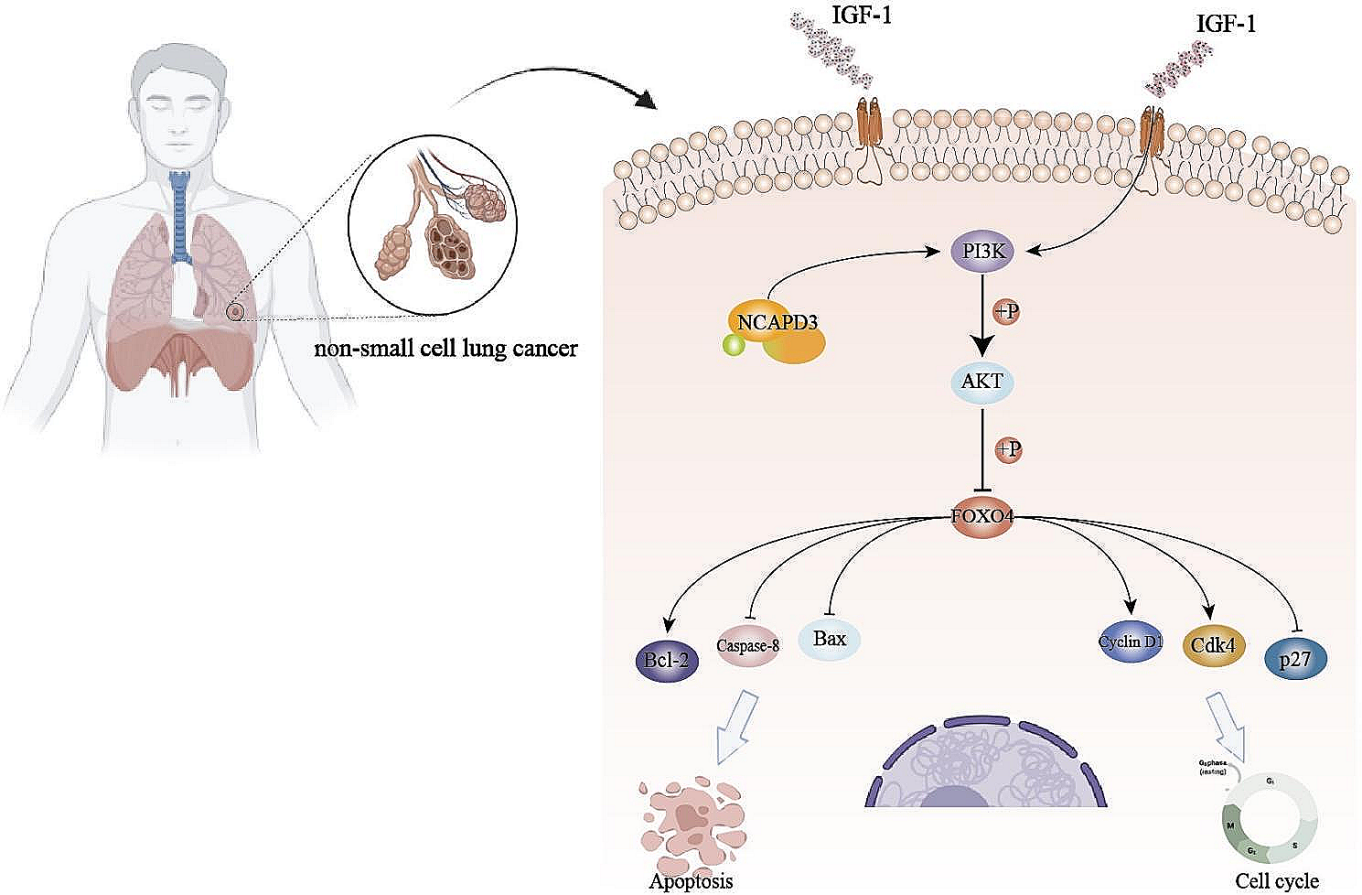
Pattern diagram for the role of NCAPD3 in NSCLC cell proliferation and apoptosis. The regulation of the PI3K/AKT/FOXO4 pathway mediates the process of NCAPD3-modulated NSCLC cell proliferation and apoptosis

Previous studies showed that stimulation of the PI3K/AKT signaling pathway is involved in tumor proliferation, angiogenesis, invasion and metastasis, and also has a significant impact on tumor resistance and chemoradiotherapy antagonism [33]. Our investigation discovered a connection between the PI3K/AKT pathway and NCAPD3, demonstrating that NCAPD3 regulates the proliferation and apoptosis of NSCLC cells by activating the PI3K/AKT pathway. Studies have shown that NCAPG, as a homo-family protein of NCAPD3, can affect the proliferation and apoptosis of liver cancer cells through the PI3K/AKT signaling pathway, which is comparable with what we discovered [31]. The mechanisms by which PI3K/AKT signaling regulates anti-apoptotic effects mainly include: (1) Enhanced expression of anti-apoptotic proteins. Pro-apoptotic proteins (Bad, Bax, etc.) and anti-apoptotic proteins (Bcl-2, Bcl-XL, etc.) are members of the Bcl-2 family. Under the action of various factors, Bad is dephosphorylated and activated, and it interferes with anti-apoptotic proteins Bcl-2 and Bcl-XL to inhibit the function of anti-apoptotic proteins. AKT is a powerful Bad kinase, which can inactivate Bad after phosphorylation, resulting in the release of anti-apoptotic proteins Bcl-2 and Bcl-XL, and exerting anti-apoptotic effects [34]. (2) Regulation of apoptotic signalling pathways, such as Caspase family proteins, which play crucial roles in initiating and executing the activity of key enzymes in apoptosis. (3) Regulation of cell survival signalling pathways, such as the NF-κB (nuclear factor kappa B) pathway and the mTOR (mammalian target of repamycin protein) pathway. (4) Inhibit the release of factors associated with mitochondrial apoptosis. The release of the mitochondrial apoptosis-related factors AIF and cytochrome C can be prevented by stimulating the PI3K/AKT signaling pathway, which then activates the apoptotic pathway to induce apoptosis [35]. (5) Affect the activity of related transcription factors. Activated AKT can directly phosphorylate related transcription factors such as Forkhead, thereby inhibiting the expression of pro-apoptotic proteins and exerting anti-apoptotic functions [36, 37]. The forkhead box protein O4 (FOXO4) is highly conserved in evolution, has the same domain as the FOX family molecules, and plays a variety of biological functions in organisms. As a downstream target, related studies have reported that FOXO is adversely modulated by the PI3K/AKT signal transduction pathway, and implicated in cell cycle arrest, DNA damage repair, and apoptosis [38]. Additionally, numeric studies have demonstrated that the binding of Bcl-6 to Bcl-XL can inhibit its expression, while the specific binding of FOXO4 to the Bcl-6 promoter can increase the expression of Bcl-6, thereby triggering apoptosis [39]. Additionally, It has been observed that high levels of FOXO4 expression cause cell cycle arrest in the G1 phase under the influence of the cell cycle inhibitory protein P27, which also causes apoptosis [40]. To identify whether FOXO4 is involved in the oncogenic phenotype of NCAPD3, we detected the expression of both p-FOXO4 and FOXO4 in the cells transfected with shNCAPD3. We found that knockdown of NCAPD3 reduced the total protein expression levels of FOXO4 in A549 and SPCA-1 cells. However, further research revealed an interesting phenomenon, where the expression levels of p-FOXO4 increased after NCAPD3 knockdown. In fact, AKT is the first kinase known to inhibit the function of FOXOs. When the PI3K/AKT signaling pathway is activated, it phosphorylates FOXOs, leading to the loss of their transcriptional activity [41]. These results indicated that PI3K-induced phosphorylation of AKT is reduced after NCAPD3 downregulation, resulting in increased p-FOXO4 activity due to the negative regulation of FOXO by PI3K/AKT, ultimately causing cell cycle arrest and increased apoptosis (Fig. 5)

The aim of this study was to elucidate the biological effects and potential molecular mechanisms of NCAPD3 on NSCLC through in vitro experiments. However, we need to acknowledge that there were some limitations in our study. Firstly, the study was conducted with a relatively small sample size, which may limit the generalizability of the findings. A larger sample size would provide more robust and representative results. Secondly, shRNA-mediated gene knockdown experiments are widely used and effective approach for studying gene function, they do have some limitations. For example, shRNA-mediated knockdown may not completely eliminate gene expression, and off-target effects can occur. In contrast, gene knockout experiments can provide more definitive evidence of gene function by completely eliminating gene expression. Additionally, experimental animal models of Xenograft tumor formation were not conducted, which need to be further explored

## Conclusions

In conclusion, our results indicate that NCAPD3 was significantly upregulated in NSCLC tissues and cells, which is associated with an unfavorable clinical prognosis. Moreover, suppression of NCAPD3 inhibits cell proliferation, invasion and migration, enhances cell apoptosis and induces G0/G1 arrest in NSCLC cells by regulation of the PI3K/AKT/FOXO4 pathway. Our findings offer a solid scientific foundation for comprehending how NSCLC progresses and indicate NCAPD3 might act as a potential biomarker for NSCLC patients

### Electronic supplementary material

Below is the link to the electronic supplementary material.

Supplementary Material 1

Supplementary Material 2

## Data Availability

The datasets generated and analyzed during the current study are available in the Gene Expression Omnibus (GEO) repository, accession number GSE249569 and link: https://www.ncbi.nlm.nih.gov/geo/query/acc.cgi?acc=GSE249569. Other data will be available from the corresponding author upon request.

## Abbreviations

NSCLC: Non-small cell lung cancer
NCAPD3: Non-SMC condensin II complex subunit D3
LUAD: Lung adenocarcinoma
LUSC: lung squamous cell carcinoma
PI3K: Phosphatidylinositol-3-kinase
AKT: Protein kinase B
FOXO4: Forkhead box O4
Bcl-2: B-cell lymphoma-2
Bax: Bcl-2-associated x protein
CDK4: cyclin-dependent kinase 4
P27: Cyclin dependent kinase inhibitor p27Kip1
IGF-1: Insulin-like growth factor-1
TNM: Tumor-node-metastasis
TCGA: The Cancer Genome Atlas
GTEx: Genotype-Tissue Expression
GO: Gene Ontology
KEGG: Kyoto Encyclopedia of Genes and Genomes
