# Supplementary material for: Knockdown of NCAPD3 inhibits the tumorigenesis of non-small cell lung cancer by regulation of the PI3K/Akt pathway
Source: BMC Cancer. 2024 Apr 2;24:408. doi: 10.1186/s12885-024-12131-x (PMC10986035; doi:10.1186/s12885-024-12131-x)
Supplement: Supplementary file 1 — Supplementary Material 1 [file 12885_2024_12131_MOESM1_ESM.docx]

**Supplementary Materials**

**Content**

[Supplementary File 1 Protocols of RNA sequencing assay and data analysis 1](#_Toc11320)

[Supplementary Table 1 Sequences of control and shNCAPD3 2](#_Toc28909)

[Supplementary Table 2 Gene-specific promers used in RT-qPCR 2](#_Toc3135)

[Supplementary Table 3 Detailed information on main reagents, consumables, and dilution ratios 3](#_Toc25897)

**Supplementary File 1. Protocols of RNA sequencing assay and data analysis**

Total RNAs were extracted from NSCLC cells with or without NCAPD3 knockdown using TRIzol Reagent (Takara, Japan). After confirming the quality and integrity of the RNA, 2ug RNAs were used for standard RNA sequencing library preparation, and the library products were finally sequenced on Novaseq 6000 sequencer (Illumina). The R package edgeR (<https://bioconductor.org/packages/edgeR>) was used to identify genes that were differentially expressed between groups. KOBAS software (version 2.1.1) was performed to implement gene ontology (GO) analysis and KEGG enrichment analysis for differentially expressed genes. The differentially expressed transcripts and genes were selected with log2 (fold change) ≥ 1 or log2 (fold change) ≤ -1 and p value < 0.05.

**Supplementary Table S1. Sequences of control and shNCAPD3**

| **Gene** | **shRNA** | **Sequence (5’ - 3’)** |
| --- | --- | --- |
| NCAPD3 | shRNA1 | ccCTCTGTGATTAGAGCACAT |
|  | shRNA2 | ccTGATTAACAGTCCTACGTT |
|  | shRNA3 | cgCTGGATCTTAGACTCGAAT |
|  | Negative control | TTCTCCGAACGTGTCACGT |

**Supplementary Table S2. Gene-specific primers used in RT-qPCR**

| **Oligonucleotides** | |
| --- | --- |
| **Gene** | **Primer sequences for RT-PCR (5’-3’)** |
| NCAPD3 | Forward: TTTCACAAACTCCTGCAAG |
|  | Reverse: CGTCATCATTGTAATGTGCGAT |
| GAPDH | Forward: GCGGGGCTCCAGAACA TCAT |
|  | Reverse: CCAGCCCCAGCGTCAAGGTG |

**Supplementary Table S3. Detailed information on main reagents, consumables, and dilution ratios**

| **REAGENT or RESOURCE** |  |  |
| --- | --- | --- |
| **Antibodies** | **Dilution ratio** | **SOURCE/IDENTIFIER** |
| Rabbit anti-NCAPD3  Mouse anti-GAPDH  Rabbit anti-Bcl-2  Rabbit anti-Bax  Rabbit anti-Caspase-8  Rabbit anti-Cyclin D1  Rabbit anti-Cdk4  Rabbit anti-P27  Rabbit anti-PI3k  Rabbit anti-AKT  Rabbit anti-p-AKT  Rabbit anti-FOXO4  Rabbit anti-p-FOXO4  Goat anti-Rabbit IgG (HRP) |  | Abnova (Catalog #H00023310-M01)  Abcam (Cat #ab8245)  Abcam (Cat #ab32124)  Abcam (Cat #ab32503)  Abcam (Cat #ab108333)  Abcam (Cat #ab134175)  Abcam (Cat #ab108357)  Abcam (Cat #ab32034)  Abcam (Cat #ab302958)  Abcam (Cat #ab8805)  Abcam (Cat #ab38449)  Abcam (Cat #ab128908)  Abcam (Cat #ab174849)  Abcam (Cat # ab6721) |
| Goat anti-Mouse IgG (HRP) |  | Abcam (Cat #ab6789) |
| **Chemicals, Peptides, and Recombinant Proteins** | | |
| Opti-MEM | Gibco | Cat #31985070 |
| Lipofectamine 2000 | biosharp | Cat #BL623A |
| TRIzol  SYBR Green qPCR master mix  RPMI 1640  DMEM  Fetal bovine serum  Penicillin-Streptomycin  PVDF membranes  RIPA lysis buffer | Takara  Thermo Fisher Scientific  Gibco  Gibco  Gibco  Tico  Merck Millipore  Beyotime | Cat #9108Q  Cat #4309155  Cat #C11875500BT  Cat #C11965500BT  Cat #FBSEU500  Cat #15140-122  Cat #IPVH00010  Cat #P00138 |
| **Software and Algorithms** |  |  |
| Image J  Graphpad Prism | Schneider et al., 2012  Graphpad Software | RRID: SCR_003070  <https://imagej.nih.gov/ij>  RRID: SCR_002798  <https://www.graphpad.com> |
