# Supplementary material for: Knockdown of NCAPD3 inhibits the tumorigenesis of non-small cell lung cancer by regulation of the PI3K/Akt pathway
Source: BMC Cancer. 2024 Apr 2;24:408. doi: 10.1186/s12885-024-12131-x (PMC10986035; doi:10.1186/s12885-024-12131-x)
Supplement: Supplementary file 2 — Supplementary Material 2 [file 12885_2024_12131_MOESM2_ESM.pdf]

Corresponds to Figure 2

Figure 2-(a)-GAPDH

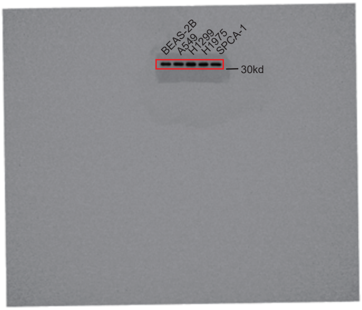

Figure 2-(a)-NCAPD3

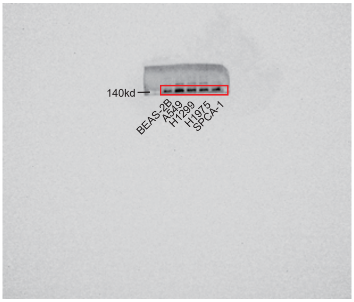

Figure 2-(d)-A549-GAPDH

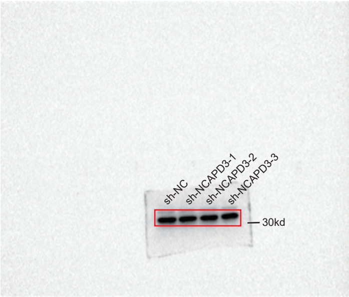

Figure 2-(d)-A549-NCAPD3

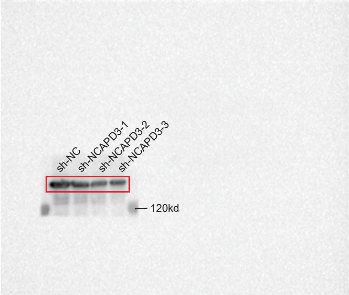

Figure 2-(d)-SPCA-1-GAPDH

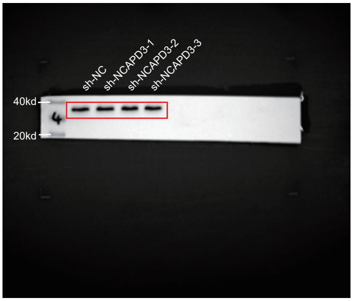

Figure 2-(d)-SPCA-1-NCAPD3

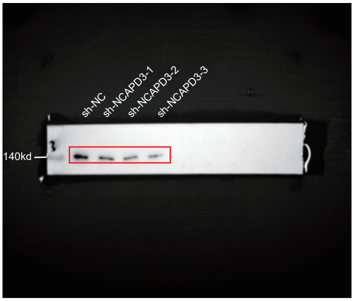

Corresponds to Figure 3 (c)

Figure 3-(c)-A549-Bcl-2

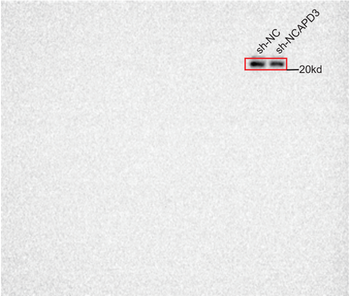

Figure 3-(c)-SPCA-1-Bcl-2

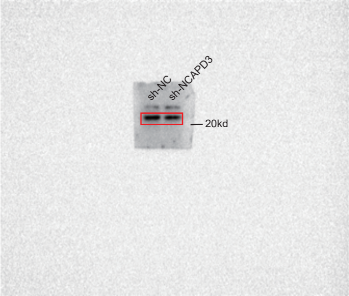

Figure 3-(c)-A549-Bax

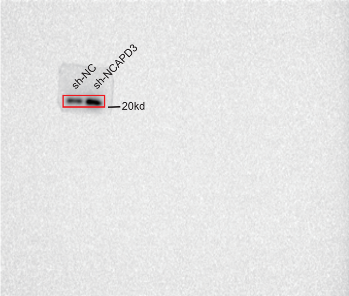

Figure 3-(c)-SPCA-1-Bax

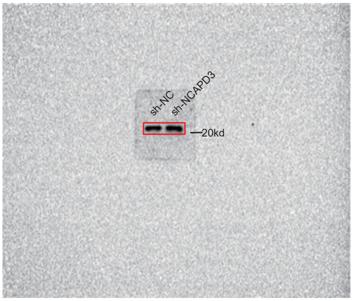

Figure 3-(c)-A549-Caspase-8

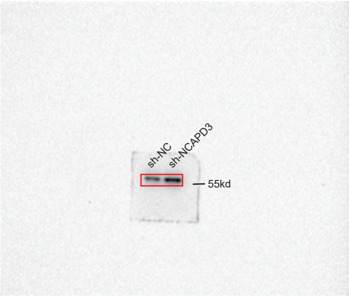

Figure 3-(c)-SPCA-1-Caspase-8

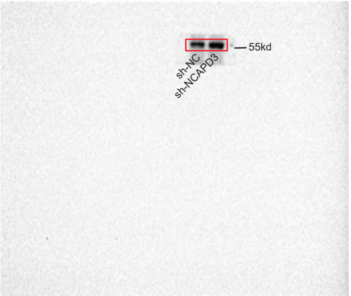

Corresponds to Figure 3(c)

Figure 3-(c)-A549-GAPDH

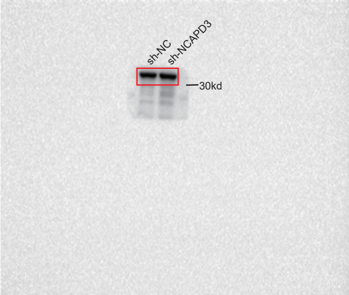

Figure 3-(c)-SPCA-1-GAPDH

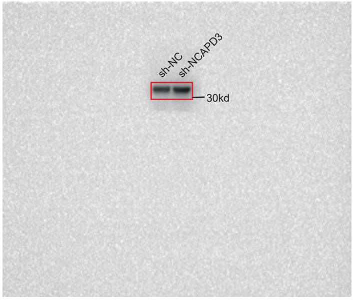

Figure 3-(c)-A549-CyclinD1

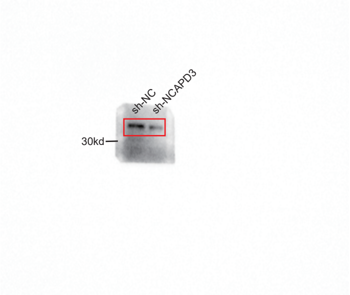

Figure 3-(c)-SPCA-1-CyclinD1

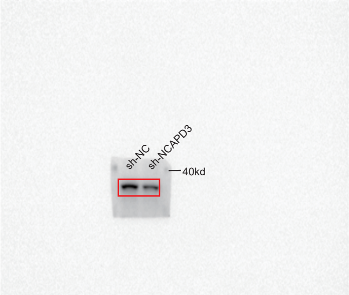

Figure 3-(c)-A549-Cdk4

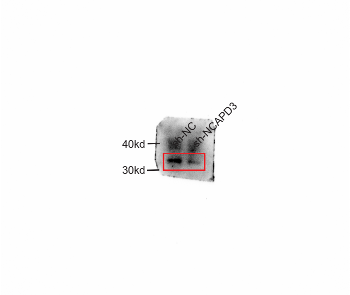

Figure 3-(c)-SPCA-1-Cdk4

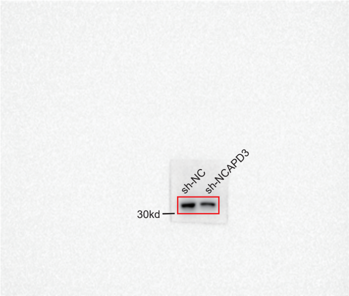

Figure 3-(c)-A549-P27

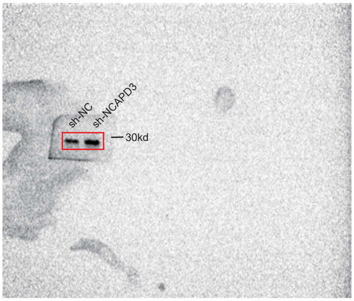

Figure 3-(c)-SPCA-1-P27

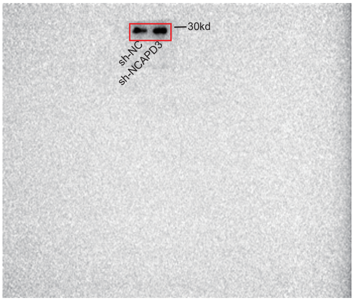

Figure 3-(c)-A549-GAPDH

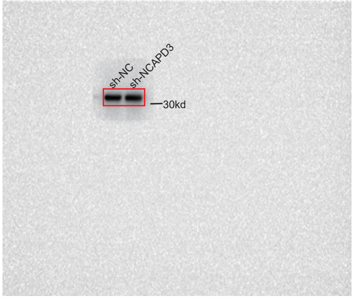

Figure 3-(c)-SPCA-1-GAPDH

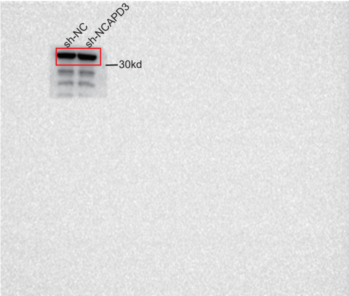

Corresponds to Figure 4 (c)

Figure 4-(c)-A549-PI3K

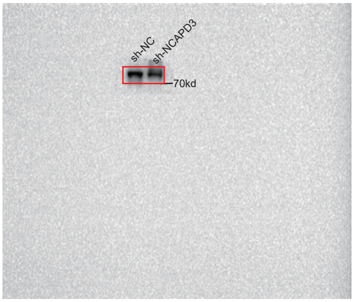

Figure 4-(c)-SPCA-1-PI3K

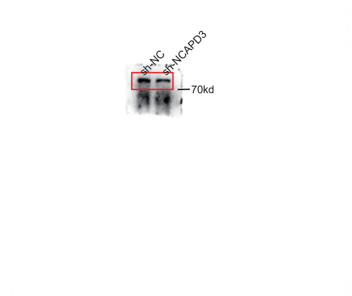

Figure 4-(c)-A549-AKT

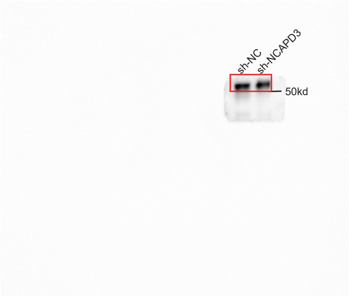

Figure 4-(c)-SPCA-1-AKT

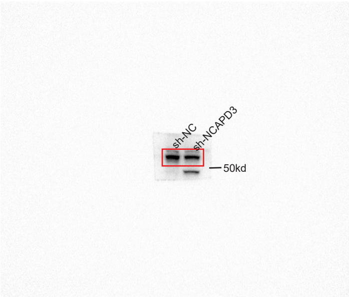

Figure 4-(c)-A549-pAKT

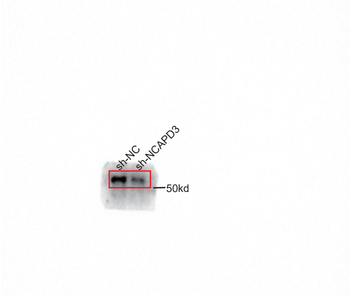

Figure 4-(c)-SPCA-1-pAKT

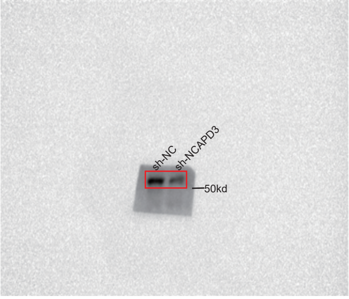

Figure 4-(c)-A549-FOXO4

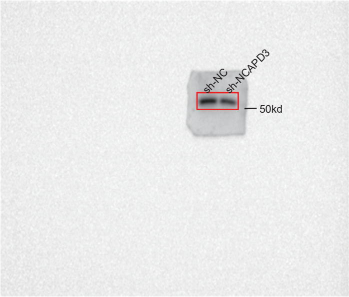

Figure 4-(c)-SPCA-1-FOXO4

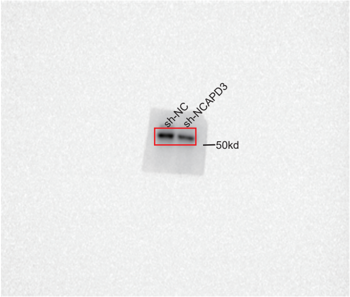

Figure 4-(c)-A549-pFOXO4

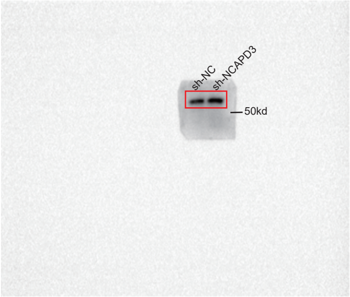

Figure 4-(c)-SPCA-1-pFOXO4

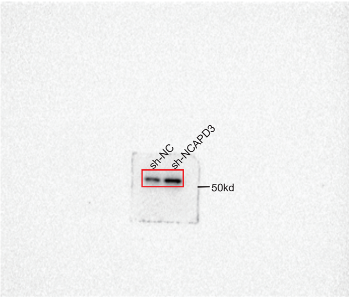

Figure 4-(c)-A549-GAPDH

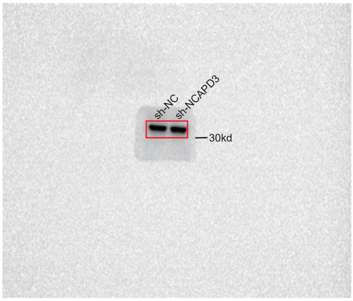

Figure 4-(c)-SPCA-1-GAPDH

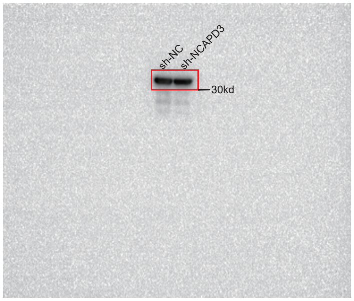

Corresponds to Figure 4 (d)

Figure 4-(d)-A549-Bcl-2

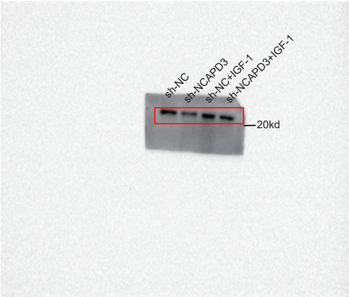

Figure 4-(d)-SPCA-1-Bcl-2

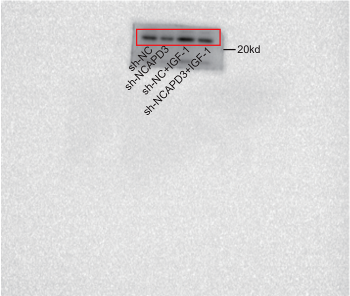

Figure 4-(d)-A549-Bax

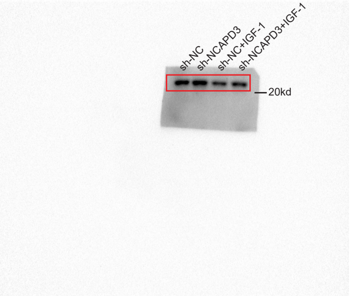

Figure 4-(d)-SPCA-1-Bax

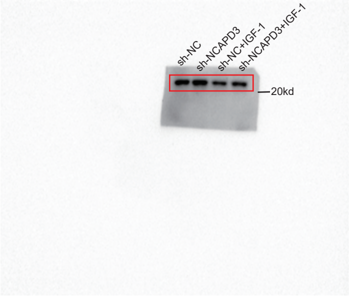

Figure 4-(d)-A549-Caspase-8

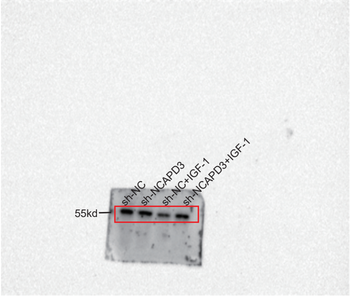

Figure 4-(d)-SPCA-1-Caspase-8

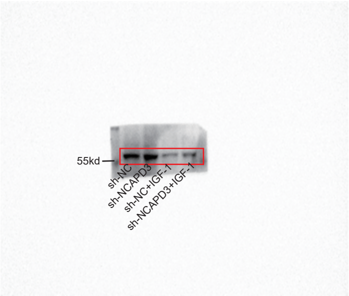

Figure 4-(d)-A549-Cyclin D1

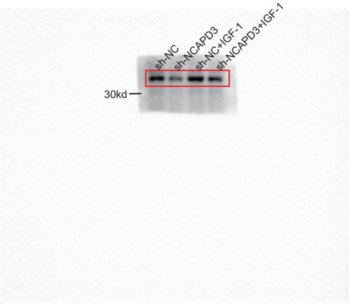

Figure 4-(d)-SPCA-1-Cyclin D1

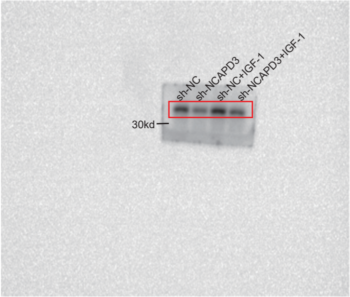

Figure 4-(d)-A549-Cdk4

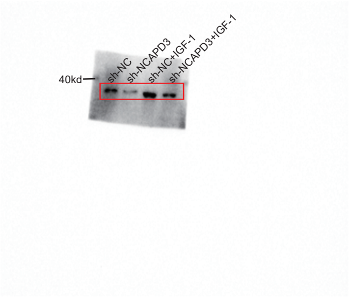

Figure 4-(d)-SPCA-1-Cdk4

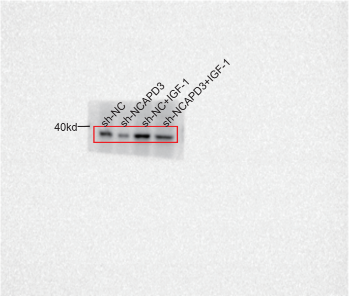

Figure 4-(d)-A549-P27

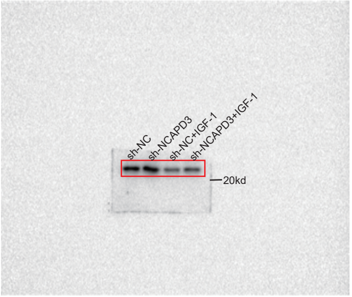

Figure 4-(d)-SPCA-1-P27

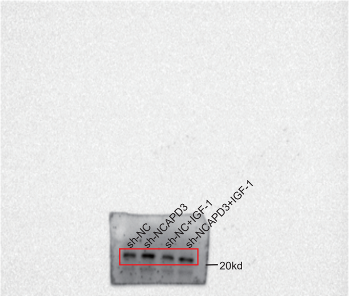

Corresponds to Figure 4 (d)

Figure 4-(d)-A549-GAPDH

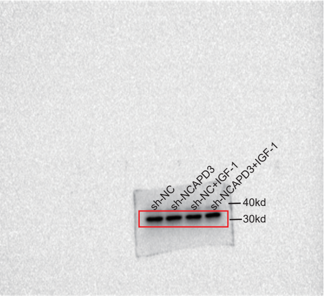

Figure 4-(d)-SPCA-1-GAPDH

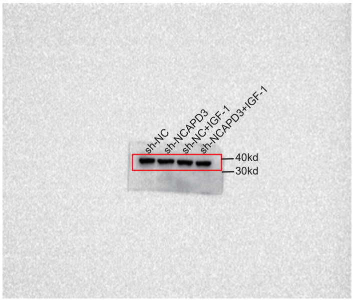

Statement:The membranes were cut prior to antibody hybridization, resulting in the absence of the full-length blot.
